# In Vitro Anthelminthic Efficacy of Aqueous Pomegranate (*Punica granatum* L.) Extracts against Gastrointestinal Nematodes of Sheep

**DOI:** 10.3390/pathogens9121063

**Published:** 2020-12-18

**Authors:** Fabio Castagna, Domenico Britti, Manuela Oliverio, Antonio Bosco, Sonia Bonacci, Giuseppe Iriti, Monica Ragusa, Vincenzo Musolino, Laura Rinaldi, Ernesto Palma, Vincenzo Musella

**Affiliations:** 1 Department of Health Sciences, University of Catanzaro “Magna Græcia”, CISVetSUA, 88100 Catanzaro, Italy; britti@unicz.it (D.B.); m.oliverio@unicz.it (M.O.); s.bonacci@unicz.it (S.B.); giuseppeiriti94@gmail.com (G.I.); palma@unicz.it (E.P.); musella@unicz.it (V.M.); 2 Department of Veterinary Medicine and Animal Productions, University of Naples Federico II, 80137 Napoli, Italy; boscoant@tiscali.it (A.B.); lrinaldi@unina.it (L.R.); 3 Department of Experimental and Clinical Medicine, University of Catanzaro Magna Græcia, CISVetSUA, 88100 Catanzaro, Italy; m.ragusa@unicz.it

**Keywords:** gastrointestinal nematodes, sheep, aqueous *Punica granatum* macerate, plant extracts, anthelmintic efficacy, chemical characterization, chromatographic analysis

## Abstract

The worldwide increased difficulty to counteract gastrointestinal nematode (GIN) infection in sheep, due to progressing anthelmintic resistance, has led to the evaluation of other alternative helminth control options, mainly from plants. The anthelmintic efficacy of an aqueous *Punica granatum* macerate was evaluated in sheep naturally infected by GIN in southern Italy. The macerate was chemically characterized by chromatographic analysis coupled with high-resolution mass spectrometry (LC/HRMS) and an aliquot was concentrated to obtain a dry extract. A part was characterized, the remaining washed with methanol to obtain an insoluble residue and methanol phase. In the methanol fraction, the quantitatively predominant gallic acid was purified to obtain the pure molecule. The three fractions thus obtained were used for in vitro studies (i.e., egg hatch test) to verify anthelmintic efficacy. For this purpose, fecal samples were collected from sheep naturally infected by GINs. Fractions were diluted in H_2_O/DMSO 0.5% at 1.00, 0.5, 0.25, 0.125, 0.05, and 0.005 mg/mL concentrations. Thiabendazole (0.25 and 0.5 mg/mL) and deionized water were used as positive and negative controls, respectively. Egg hatch test results indicated that all fractions caused a significant (*p* < 0.05) egg hatch inhibition within 48 h of exposure highlighting a high (>82%) efficacy in vitro at all tested doses. Maximal egg hatching inhibition effect was exhibited by the methanol fraction (99.3% and 89.3% at 1 and 0.005 mg/mL concentrations), followed by the insoluble residue and gallic acid (94.7% and 85.3% and 94.0% and 82.7% at 1 and 0.005 mg/mL, respectively). The current study validated the anthelmintic potential of traditional *P. granatum* macerate against GIN infection in sheep, thus highlighting the role of gallic acid as principal component and justifying a need to undertake further in vivo studies on these ethno-veterinary remedies.

## 1. Introduction

Infections with parasites continues to represent a serious challenge to health, welfare, productivity, and reproduction of grazing ruminants throughout the world [1]. Helminths infect livestock and cause the most significant losses to food production globally [2]. In particular, gastrointestinal nematode (GIN) infection, caused by different genera of nematodes (e.g., *Teladorsagia*, *Haemonchus*, *Trichostrongylus*, and *Oesophagostomum*) remains one of the main constraints to small ruminant production in southern Italy [3].

Since the mid-1960s, GIN control in livestock has heavily relied on anthelmintics. At the time of first registration, all anthelmintics used in livestock were very effective, typically reducing susceptible worm burdens by up to 99% [4]. Currently, their control is still primarily relied on the use of chemical anthelmintic drugs [5] and the availability of highly efficacious anthelmintic drugs has significantly contributed to reducing the economic burden of GIN infections [6,7]. However, after years of intensive use to optimize animal productivity, the widespread appearance of resistant parasites was inevitable [8]. Anthelmintic resistance (AR) has become a major and growing problem on small ruminant farms in many countries, EU included [9].

The maintenance of anthelmintic efficacy is important to ensure high levels of production and animal welfare [10] and the expanding development and diffusion of AR in nematode populations imposes the need to explore and validate novel solutions (or to re-discover old knowledge) for a more sustainable control of GIN in ruminants [5]. All the above has provided an impetus for the research aimed at the development of alternative control methods [11]. In many countries of the world, in particular in Asia and Africa [12,13,14] and in some European countries [15], plants have been used in local traditions for parasite control in small ruminants. These traditions have survived in some area of Italy [16,17], in particular in southern regions [18,19], where some aqueous plant-based macerates, including pomegranate (*Punica granatum* L.), are used by several farmers and pastoralists.

*P. granatum* plant is native in Asian countries and it has been cultivated and naturalized over the whole Mediterranean region since ancient times. This botanical species has been the subject of study as a medicinal agent with wide variety therapeutic indications [20] because different parts, such as bark, leaves, fruits and fruit rind, have medicinal significance [21]. For these reasons *P. granatum* has been used in various region in traditional medicine as a treatment for many diseases, such as repellent against medically important mosquito vectors, parasitic worm infections, and against protozoan parasites infections [22,23,24]. The anthelmintic activity of pomegranate is well known by the literature [25,26,27,28]. This activity seems to be mainly due to the pelletierine, a molecule belonging to the class of the alkaloids, present in the bark of the root of *P. granatum* [29,30]. However, pomegranate extracts are phyto-complexes characterized by a wide range of different compounds. Thus, the aims of the present study were to evaluate the in vitro anthelmintic efficacy of an aqueous *P. granatum* macerate against GINs of sheep in the Calabria region of southern Italy and to identify other molecules of *P. granatum* with anthelmintic action.

## 2. Results

### 2.1. Chemical Characterization

As preliminary study, different extraction techniques and solvents were used to usefully fractionate the aqueous pomegranate macerate. Each test was performed on 5 mL of aqueous macerate, corresponding to ≈300 mg of dry extract. The obtained fractions were qualitatively compared by TLC (eluent phase DCM/MeOH *v*/*v* 8:2) Firstly, apolar organic solvents, such as DCM and AcOEt, were used in a liquid/liquid extraction (LLE). While DCM LLE was quite ineffective, AcOEt led to the recovery of 10% *w*/*w* fraction. In addition, washing and filtration of dried extract was performed using both EtOH and MeOH. Each washing resulted in a qualitatively similar fraction respect to the AcOEt LLE extraction, but with a recovery efficiency of 30% *w*/*w* for EtOH and 70% *w*/*w* for MeOH, respectively. Consequently, the macerate was fractionated, as described in the experimental part, according to the solubility in methanol. Before fractionation, the ethanol amount present in the aqueous macerate was quantified as 2.994 mg/mL. The two obtained fractions, namely, the methanol fraction A and the insoluble residue B, were analyzed by LC-HRMS and compared with the aqueous macerate.

**Figure 1:**
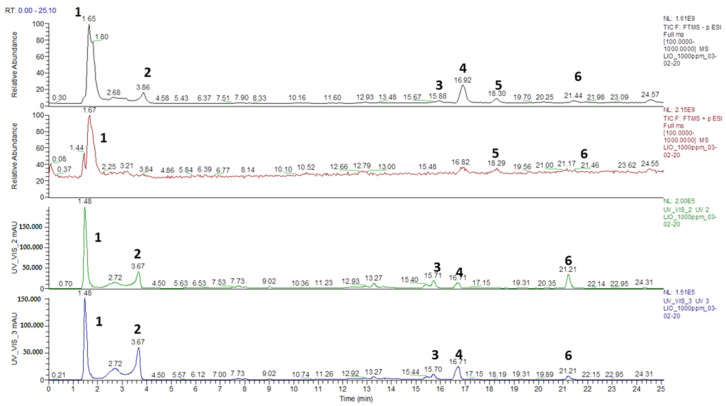
Full scan LC-HRMS analysis of the total dry extract, as registered in ESI(-), ESI(+) UV/VIS (240 nm) e UV/VIS (280 nm) detection modes, respectively.

The chromatographic characterization of the whole macerate is showed in Figure 1. The analogues LC/HRMS analysis for the three fraction A, and B are reported in Supplementary Materials.

It is clear that the ESI(-) ionization mode provides the widest number of information on the chemical composition of the mixture. Mass spectra are complementary to UV/VIS analyses. The chromatogram can be divided into two distinct regions according to the retention times (r.t.).

The first region, with r.t. between 0 and min, is composed by the molecules not strongly retained by the stationary phase, such as simple and complex sugars (compounds **1**, **2**, Figure 1).

The second region between 15 and 25 min r.t. was populated by the characteristic peaks of phenolic acids and elagiotannins (compounds **3**, **4**, **5** and **6**, Figure 1). For each representative peak shown in Figure 1, MS/MS ESI analysis was performed in order to achieve their full identification. The characterization, with the *m*/*z* values (obtained for LC/HRMS, ESI(-)) of each component together with their structural identification, is shown in Table 1.

The peak identified as (1) is a mixture of tartaric acid, glucuronic acid, mannitol, and a small percentage of an elagitannin complex (2,3-(S)-hexahydroxyphenyl-D-glucose).

The same peak was found, as unique component, in the chromatogram of the insoluble residue fraction B (See Supplementary Materials). As fraction B represents the 30% *w*/*w* of the dry extract, it can be concluded that the extract is composed for the 30% *w*/*w* of a mixture of simple organic acids and sugars.

**Figure 2:**
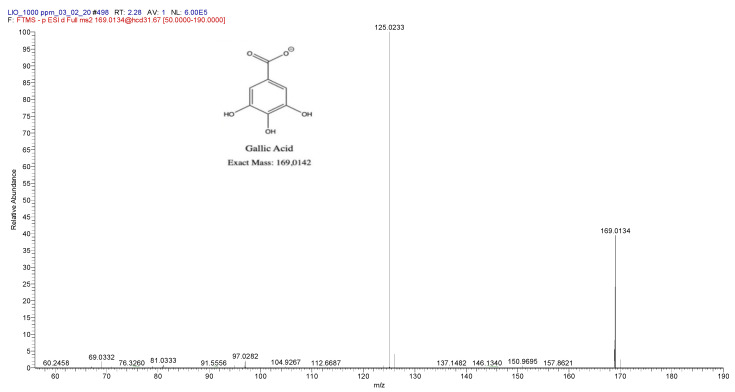
HRMS/MS/MS spectrum of gallic acid C, registered in ESI(-) ionization mode.

The peak identified as (2) in Figure 1 represented the main component in the methanol fraction A (See Supplementary Materials). It was isolated, quantified respect to the total dry extract, and characterized by ESI(-)-HRMS and ^1^H-NMR. Figure 2 shows its HRMS/MS/MS spectrum, which unequivocally identifies gallic acid. The comparison with the HRMS/MS spectrum of a reference standard confirmed the structural hypothesis. Therefore, it has been observed that the dry extract is composed of about 10% *w*/*w* by gallic acid.

The remaining part of chromatogram was composed by two elagiotanninic derivatives valoneic acid and felligridine J (peak 3, Figure 1), siringic acid (peak 4, Figure 1), ellagic acid, and ducheside A (peak 6, Figure 1) and an unknown molecule corresponding to peak 5. All these components composed, together with gallic acid C, the methanol fraction A (see Supplementary Materials). Overall, the mixture of these components represents 60% *w*/*w* of the dry extract. Pelletierine was not found in any fractions nor in the dry extract.

### 2.2. Parasitological Studies

#### 2.2.1. Coprocultures

The genera of nematodes present were: *Trichostrongylus* spp. (43%), followed by *Haemonchus contortus* (23%), *Teladorsagia* spp. (21%), *Chabertia ovina* (7%), and *Cooperia* spp. (6%).

#### 2.2.2. Egg Hatch Test

EHT efficacy profile of *P. granatum* fractions, as percentage of eggs unhatched (mean of triplicates), the positive control (TBZ) and negative control (deionized water/DMSO 0.5%) are shown in Table 2.

The results showed 6.7% of eggs in the negative control (deionized water), 96.7% of eggs in the first positive control (0.25 mg/mL of TBZ), and a 99.3% of eggs in the second positive control (0.5 mg/mL of TBZ). As regards the results of the groups with methanol (A), insoluble residue (B), and gallic acid (C) at all concentrations, percentages of >82%, significantly higher (*p* < 0.001) than the positive control, have been obtained The results also showed that at each concentration, group A always had a percentage of unhatched eggs higher than group B and C (*p* < 0.005).

PH analysis for all fractions did not show any change in variation of acidity or basicity. The measured pH did not deviate from neutrality, showing a range between 6.9 and 7.1.

## 3. Discussion

Anthelmintic resistance in ruminants is a severe and worsening problem worldwide and its development is a natural evolutionary process that is difficult to prevent if anthelmintics are overused/misused in the farm [31]. This phenomenon and risks associated with the presence of anthelmintic drug residues in environment and animal origin foods, have encouraged the search for alternative molecules from plants [32]. An additional constraint in the anthelmintic use in livestock comes from the consumer and the growing demand for food drug-free products [33,34]. In this contest, the consumers consider the respect for the environment an essential principle of breeding and often choose products carefully from animals raised with natural systems without the use of drugs [35]. Therefore, further researches are needed to reduce the use of anthelmintics through the development of alternative approaches [36]. Several studies showed the potential of plants rich in tannin on nematodes control [37,38] and, in particular, some in vitro studies highlight the inhibition of tannins contained in plants on the hatching of GIN eggs [39]. Tannins have also been shown to interfere with oxidative coupled phosphorylation and block ATP synthesis in *Haemonchus contortus* [40].

The anthelmintic efficacy of plant-based alkaloids was confirmed by Wang et al. (2010) [41]. Moreover, in vitro studies report that these compounds, contained in *P. granatum*, have antiprotozoal [42,43], anticestoda, and antinematoda properties in various species [44], including humans [45]. An improvement in histopathological picture of the jejunum, as well as the antioxidant status protects the host tissue from injuries induced by parasites was also highlighted [38]. These properties should be ascribed mainly to phenolic compounds [46,47].

In the current study all fractions produced a significant ovicidal activity against sheep GINs at the all concentrations tested in vitro. The exhibited ovicidal effectiveness is comparable with the TBZ drug. These findings are in line with the in vitro and in vivo studies done by Hassan et al. (2020) [28] and Anjos et al. (2016) [48] that confirmed the efficacy of the *P. granatum* extract against *Haemonchus contortus* in goats and *Haemonchus* spp. and *Cooperia* spp. in cattle, respectively. The ovicidal activities (<82%) detected in our study were higher than those reported in vitro by Aliyi et al. (2020) that reported moderate anthelmintic activity of *P. granatum* at the lowest concentrations against GIN in sheep (49.33 at 0.1 mg/mL, 60.67 at 0.25 mg/mL, and 72.67 at 0.5 mg/mL concentration) [27]. However, in their study Aliyi et al. (2020) [27] had used only peels crude extract of the pomegranate, while the aqueous macerate used in this study was a phytocomplex obtained from fruits and fruit rind. The exhibited anthelmintic effect might be attributed to the synergistic action of the components highlighted with chemical characterization. In particular, a synergistic effect due to the presence of different tannin-derivatives and phenolic acids was certainly present in the A fraction. Regarding the B fraction, a significant contribution of tartaric acid and ellagitannin to this activity can be assumed, while the activity of the C fraction as a pure molecule is quite surprising. This study validated the anthelmintic potential of traditional *P. granatum* macerate against GIN in naturally infected sheep, thus disclosing the role of gallic acid as principal component and justifies a need to undertake further in vivo studies on this macerate, because the concentrations of active substances used in vitro do not always correspond to in vivo bioavailability.

## 4. Materials and Methods

### 4.1. Aqueous Macerate Preparation

Aqueous pomegranate (*Punica granatum*) macerate used for this study was a traditional mixture used in the Calabria region of southern Italy against GINs of sheep.

This macerate was prepared by a Calabrian elderly breeder by macerating ripe pomegranates (fruits and fruit rind) in spring water for at least 10 months. After this time the mixture was filtered with a cloth and was ready for its use. This preparation was carried out according to ancient ethno-veterinary recipes handed down for centuries from generation to generation.

### 4.2. Chemical Characterization

The macerate was fractionated and each fraction was analyzed using the LC/MS-ESI. A 75 mL aliquot of pomegranate macerate has been concentrated in a speedvac and freeze-dried to obtain 4230 g of total dry extract.

A small portion of the extract (15%) was retained for analysis, while the main part was washed on a porous septum with methanol to obtain an insoluble residue and a methanol phase. The methanol-soluble portion was evaporated under reduced pressure to achieve 2.61 g of dry fraction (A, 70% *w*/*w*); the insoluble residue was dried using a vacuum-evaporator and weighed to obtain 0.90 mg (B, 30 *w*/*w*). The methanol fraction, analyzed by TLC (thin layer chromatography), appeared as a complex mixture of components, one of which was quantitatively predominant. The mixture was then further purified by flash chromatography (eluent phase dichloromethane/methanol 8:2) to separate the main component, obtaining 330 mg of a pure molecule (gallic acid) (13% of the methanol extract, about 10% of the total dry extract). All obtained fractions were then characterized by LC/HRMS in order to identify their components. The pure molecule was identified by direct infusion ESI-HRMS, then compared with a reference standard, while ^1^H-NMR spectrum was compared to the one reported in the literature (from spectral Database for Organic compounds SDBS).

Chromatography was performed using a Thermo Scientific (Rodano, MI, Italy) Dionex Ultimate 3000 RS, injecting directly into a Thermo Scientific Hypersil Gold C18 column (50 × 2.1 mm, 1.9 µm particle size), equilibrated in 95% solvent A (0.1% aqueous solution of formic acid) and in 5% solvent B (methanol). The column and autosampler temperatures were maintained at 24 °C and 20 °C, respectively. The elution flow rate was 200 µL/min, the column was equilibrated for 2 min with 5% solvent B, after increasing solvent B concentration from 5 to 100% in 45 min, remain for 2 min in isocratic conditions and then decreasing solvent B from 100% to 5% in 8 min and remain for other 3 min in isocratic conditions. The total run time, including column wash and equilibration was 60 min. For High Resolution Mass Spectrometry (HRMS) a Thermo Scientific Q-Exactive™ (Rodano, MI, Italy) mass spectrometer was operated using electrospray with both negative and positive polarities, at 70,000 resolving power (defined as FWHM at *m*/*z* 200), IT 100 ms, and ACG target = 3 × 10^6^, by full scan analysis (mass range 100 to 1000 amu). Source conditions were spray voltage 3.0 KV, sheath gas: 20, arbitrary units, Auxiliary gas: 8, probe heater temperature: 280 °C; capillary temperature: 320 °C; S-Lens RF Level: 50. Prior to the beginning of the analysis, the instrument was daily calibrated by “Thermo ESI negative ion calibration solution” containing sodium dodecylsulfate (*m*/*z* 265.145), sodium taurocholate (*m*/*z* 514.278) and Ultramark 1621 (*m*/*z*: 1279.982, 1379.974, 1479.967, 1579.958, 1679.952, 1769.990). Moreover, a daily analysis of a standard reference solution of gallic acid (50 mg/L) was performed in order to assess the LC-HRMS method stability.

Quantification of ethanol in the macerate was performed through head-space gas chromatography on a GC-Trace 1310 Thermo Scientific equipped with a TG-624 (30 m × 0.32 mm × 0.18 μm) and a flame ionization detector (FID). The samples were injected via a Triplus RSH Head-space system with a 1 mL standard injection valve.

Chromeleon software was used for the operation of the chromatograph and acquisition and processing of data. The GC system was supplied with H2 (35 mL/min) from a NM-H2-500 generator (Thermo Scientific), Air (flow rate 350 mL/min), and N2 (5 mL/min).

The head-space was set to 80 °C for 5 min, then the sample was transferred to the GC system. The GC inlet and syringe were heated to 150 °C and 100 °C, respectively. The gas chromatography system was operated under the following conditions: oven temperature at 40 °C for 6 min and then heated to 140 °C, heating rate 20 °C/min; detector temperature 180 °C.

### 4.3. Parasitological Study

#### 4.3.1. Recovery of GIN Eggs

GIN eggs were recovered from samples collected directly from the rectal ampulla of naturally infected sheep in a farm located in southern Italy. The fecal samples were processed within 2 h of collection by using the egg recovery technique as described by Coles et al. (1992) [49] and Bosco et al. (2020) [36].

Firstly, fecal samples were homogenized and filtered under running water through sieves with a mesh size of 125, 63, and 38 µm in order to separate the eggs from the feces. Next, the GIN eggs retained on the last sieve were washed and centrifuged for 3 min at 170× *g* with distilled water, after which the supernatant was discarded. In the end, centrifugation was performed using 40% sugar solution to float the eggs which are then isolated in new tubes, mixed with distilled water and then centrifuged two more times in order to remove pellets and to get aqueous solution with eggs.

Eggs were examined microscopically to record if embryonation had not begun. Ten aliquots of 0.1 mL were taken, and the number of eggs counted [50]. The mean number of eggs counted in these aliquots was 150 per 0.1 mL of egg suspension.

#### 4.3.2. Coprocultures

In order to identify the GIN genera, larval cultures were performed, following the protocol described by the Ministry of Agriculture, Fisheries, and Food [51]. Developed third-stage larvae (L3) were identified using the morphological keys proposed by van Wyk and Mayhew (2013) [52]. Identification and percentages of each nematode genera were conducted on 100 L3; if a sample had 100 or less L3 present, all larvae were identified. Thus, on the total number of larvae identified, it was possible to give the percentage of each genus.

#### 4.3.3. Anthelmintic Efficacy

Egg hatch test (EHT) was performed to evaluate the in vitro anthelmintic efficacy of the three fractions. The EHT procedure followed that recommended by the World Association for the Advancement of Veterinary Parasitology (WAAVP) [49]. Each fraction (A, B and C) was analyzed by three replicates and tested at different concentrations compared to the negative control (deionized water/0.5% DMSO without anthelmintic) and the positive controls (thiabendazole).

The protocol to prepare thiabendazole (TBZ, Sigma, Saint Louis, MO, USA) solution used deionized water with a neutral pH.

A stock solution of thiabendazole was prepared by dissolving the pure compound in dimethyl sulfoxide (DMSO), subsequent dilutions were made in deionized water with a neutral pH, and following the protocol described by Von Samson-Himmelstjerna et al. (2009) [53].

The final concentrations in the EHT were prepared by adding 10 µL of each TBZ solution into 1.99 mL of a suspension with approximately 150 eggs/mL in water. The final TBZ concentrations used in this study were 0.2 and 0.5 mg/mL.

The A, B, and C fractions were diluted in deionized water/DMSO 0.5% at the concentrations of 1mg/mL, 0.5 mg/mL, 0.125 mg/mL, 0,05 mg/mL, and 0.005 mg/mL).

The 24-well tissue culture test plates (Corning Incorporated, Life sciences, Salt Lake City, UT, USA) were incubated for 48 h at 25 °C. The incubation was then terminated by adding 10 µL of Lugol’s iodine solution to each well. After 48 h, at least 100 eggs (dead, embryonated) and hatched first-stage larvae in each well were counted under a microscope (Leica, Wetzlar, Germany, 20×).

The pH of each extract was measured using a pH meter (Hanna instruments Hi-223 calibration check microprocessor pH meter). All pH readings were conducted on every working solution in triplicate.

#### 4.3.4. Statistical Analysis

The arithmetic mean eggs per gram of feces (EPG) and standard deviation (SD) were calculated for the different A, B, and C fractions were analyzed using a one-way ANOVA with post hoc Fisher’s least significant difference (LSD) for more intra-group analysis and not only between the groups. Statistical analysis was carried out using STATA v. 10.0 software (Stata Corp., Lakeway, TX, USA).

## 5. Conclusions

In southern Italy, some breeders and shepherds continue to use traditional plants mixtures to treat GIN infections in sheep and it can be asserted that some of these are effective. In particular, the significant in vitro ovicidal activity of aqueous *P. granatum* macerate fractions showed in the present study, highlighted the anthelmintic potential of this ethno-veterinary remedy for the control of GINs in the sheep. However, further in vivo studies are needed to confirm the obtained results and evaluate the therapeutic potential and future applicability.

## Figures and Tables

**Table 1:** Chemical characterization results of total dry extract.

Peak LC-MS	*m*/*z* Theoretical	*m*/*z* Measured	Molecular Formula	Analyte
(1)	149.0092	149.0081	C_4_H_5_O_6_	Tartaric acid
	181.0718	181.0711	C_6_H_13_0_6_	Mannitol
	193.0354	193.0347	C_9_H_9_O_7_	Glucuronic acid
	481.0697	481.0626	C_20_H_17_O_14_	2,3-(S)-hexahydroxyphenyl-D-glucose
(2)	169.0142	169.0134	C_7_H_5_O_5_	Gallic acid
(3)	288.9990	288.9992	C_13_H_5_O_8_	Phelligridin J
	469.0049	469.0050	C_21_H_9_O_13_	Valoneic acid dilattone
(4)	197.0455	197.0449	C_9_H_9_O_5_	Syringic acid
(5)	-	186.1129	C_13_H_14_O	unknown
(6)	300.9990	300.9991	C_14_H_5_O_8_	Ellagic acid
	447.0642	447.0573	C_20_H_15_O_12_	Ducheside A

**Table 2:** Percentage of eggs unhatched (mean of triplicates) and Standard Deviation (SD) after treatment at various concentrations with pomegranate macerate fractions: methanol (A), insoluble residue (B), gallic acid (C), thiobendazole (TBZ) and deionized water/DMSO 0.5%.

Concentration (mg/mL)	Methanol A	Insoluble Residue B	Gallic Acid C	TBZ (Positive Control)	Deionized Water/DMSO 0.5% (Negative Control)
0.005 mg/mL	89.3 ± 2.5 ^a^*	85.3 ± 1.5 ^b^	82.7 ± 2.1 ^b^		6.7 ^c^ ± 0.6
0.05 mg/mL	94.0 ± 2.6 ^a^	89.7 ± 1.5 ^b^	86.3 ± 1.1 ^b^		
0.125 mg/mL	96.0 ± 3.0 ^a^	90.7 ± 2.1 ^b^	90.7 ± 0.6 ^b^		
0.25 mg/mL	97.7 ± 1.5 ^a^	93.3 ± 4.2 ^b^	91.3 ± 1.5 ^b^	96.7 ^a^ ± 1.2	
0.5 mg/mL	98.0 ± 1.0 ^a^	94.3 ± 3.5 ^b^	93.0 ± 1.0 ^b^	99.3 ^a^ ± 0.6	
1 mg/mL	99.3 ± 0.6 ^a^	94.7 ± 3.8 ^b^	94.0 ± 2.0 ^b^		

* different letters highlight statistical differences between groups ^a, b^ (*p* < 0.05), ^c^ (*p* < 0.001).

## Supplementary Materials

The following are available online at https://www.mdpi.com/2076-0817/9/12/1063/s1, Figure S1: Full LC-HRMS ESI(-) of methanol fraction A; Figure S2: Full LC-HRMS ESI(-) of insoluble residue fraction B; Figure S3: HRMS ESI(-) of gallic acid fraction C; Figure S4: HRMS/MS ESI(-) of gallic acid fraction C; Figure S5: Full LC-HRMS ESI(-) of peak 1; Figure S6: HRMS/MS ESI(-) of peak 1; Figure S7: Full LC-HRMS ESI(-) of peak 2; Figure S8: HRMS/MS ESI(-) of peak 2; Figure S9: Full LC-HRMS ESI(-) of peak 3; Figure S10: HRMS/MS ESI(-) of peak 3; Figure S11: Full LC-HRMS ESI(-) of peak 4; Figure S12: HRMS/MS ESI(-) of peak 4; Figure S13: Full LC-HRMS ESI(-) of peak 5; Figure S14: HRMS/MS ESI(-) of peak 5; Figure S15: Full LC-HRMS ESI(-) of peak 6; Figure S16: HRMS/MS ESI(-) of peak 6.
