# Supplementary material for: In Vitro Anthelminthic Efficacy of Aqueous Pomegranate (Punica granatum L.) Extracts against Gastrointestinal Nematodes of Sheep
Source: Pathogens. 2020 Dec 18;9(12):1063. doi: 10.3390/pathogens9121063 (PMC7766728; doi:10.3390/pathogens9121063)
Supplement: Supplementary file 1 [file pathogens-09-01063-s001.pdf]

pathogens

Supplementary data

In Vitro Anthelminthic Efficacy of Aqueous Pomegranate (*Punica granatum* L.) Extracts against Gastrointestinal Nematodes of Sheep

Fabio Castagna [1,*], Domenico Britti [1], Manuela Oliverio [1], Antonio Bosco [2], Sonia Bonacci [1], Giuseppe Iriti [1], Monica Ragusa [3], Vincenzo Musolino [1,*], Laura Rinaldi [2], Ernesto Palma [1,†] and Vincenzo Musella [1,†]

1 Department of Health Sciences, University of Catanzaro "Magna Græcia", CISVetSUA, 88100 Catanzaro, Italy; britti@unicz.it (D.B.); m.oliverio@unicz.it (M.O.); s.bonacci@unicz.it (S.B.); giuseppeiriti94@gmail.com (G.I.); palma@unicz.it (E.P.); musella@unicz.it (V.M.)
2 Department of Veterinary Medicine and Animal Productions, University of Naples Federico II, 80137 Napoli, Italy; boscoant@tiscali.it (A.B.); lrinaldi@unina.it (L.R.)
3 Department of Experimental and Clinical Medicine, University of Catanzaro Magna Græcia, CISVetSUA, 88100 Catanzaro, Italy; m.ragusa@unicz.it
* Correspondence: fabiocastagna@unicz.it (F.C.); v.musolino@unicz.it (V.M.)
† These authors equally contributed in this work.

Received: 12 November 2020; Accepted: 16 December 2020; Published: 18 December 2020

Figure S1. Full LC-HRMS ESI(-) of methanol fraction A

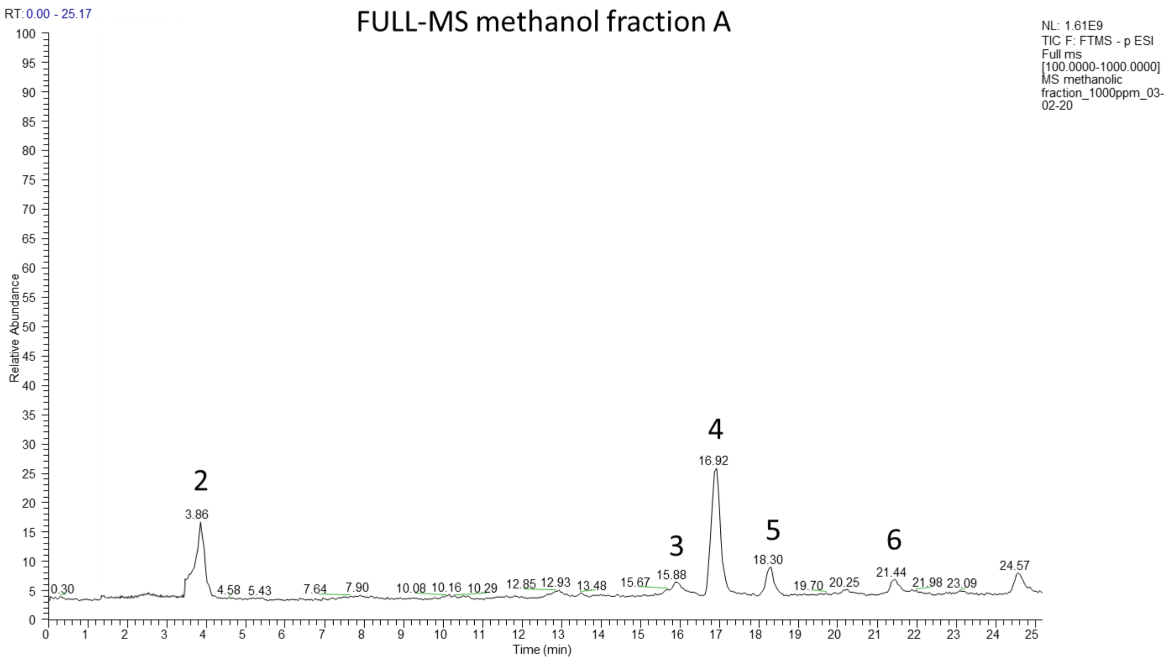

Figure S2. Full LC-HRMS ESI(-) of insoluble residue fraction B

FULL-MS insoluble residue B

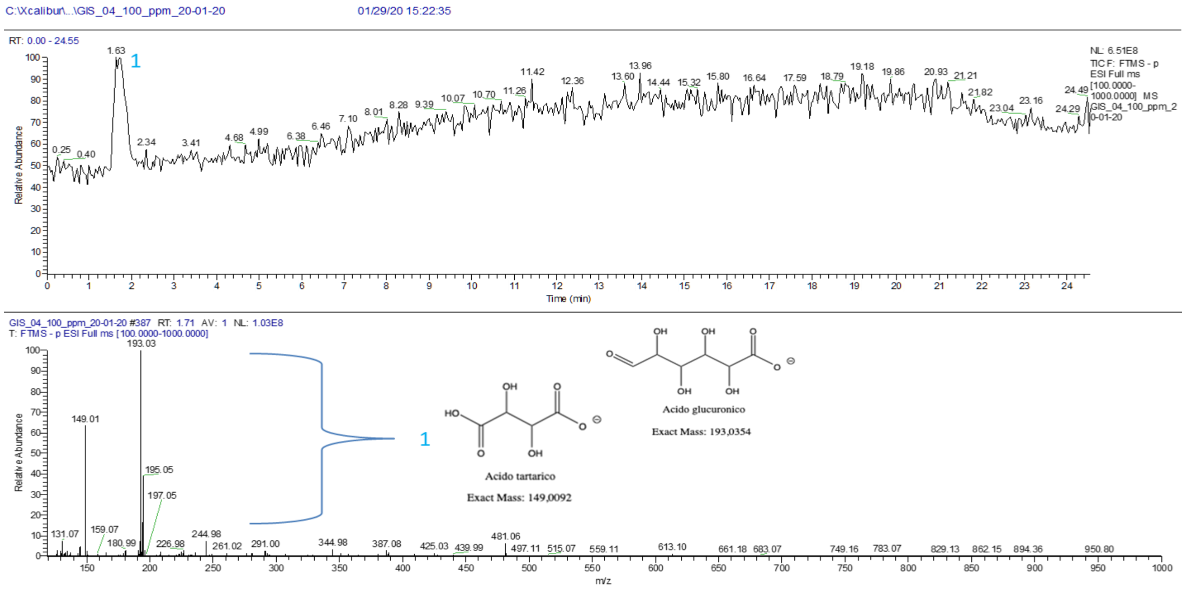

Figure S3. HRMS ESI(-) of gallic acid fraction C

ESI(-)/HRMS of gallic acid C

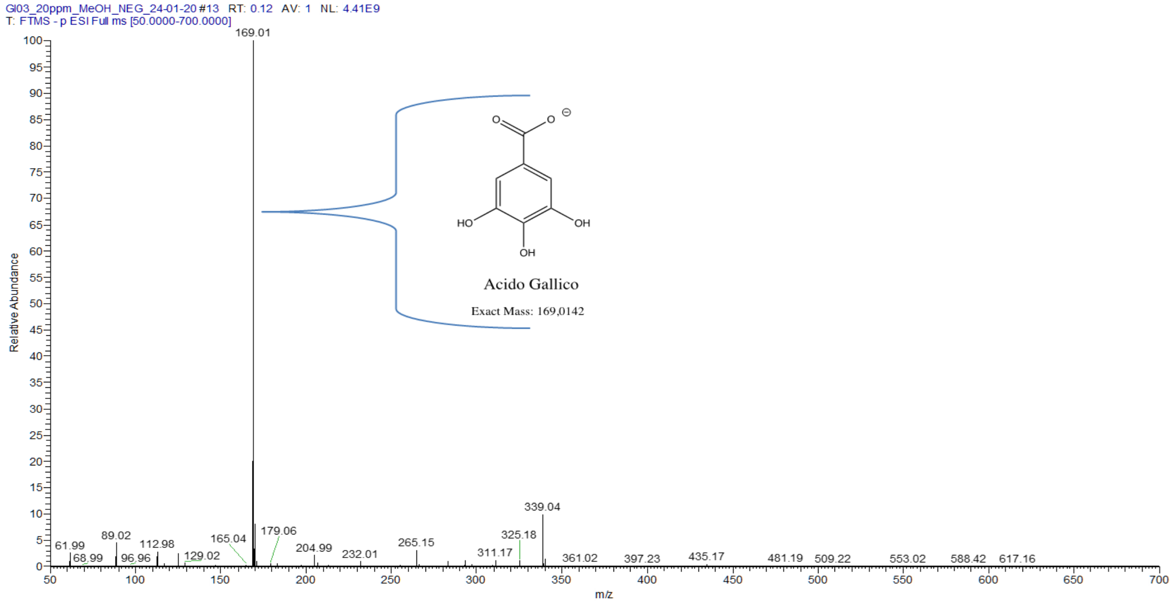

Figure S4. HRMS/MS ESI(-) of gallic acid fraction C

MS/MS gallic acid C

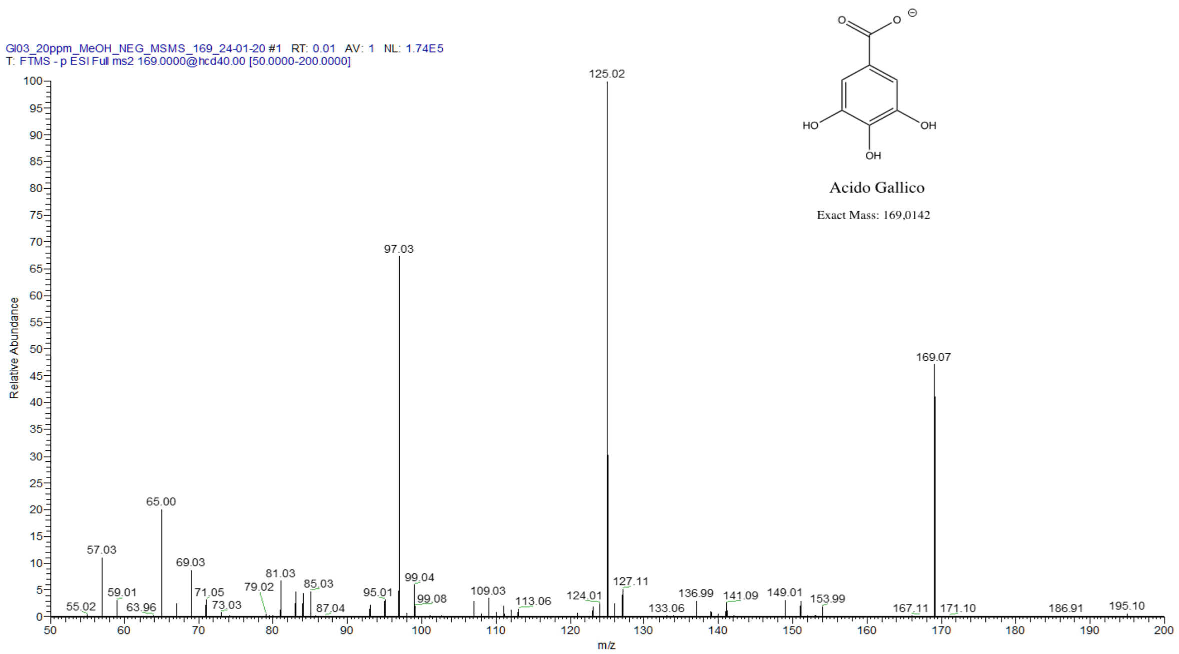

Figure S5. HRMS/MS ESI(-) of gallic acid fraction C

FULL-MS peak 1

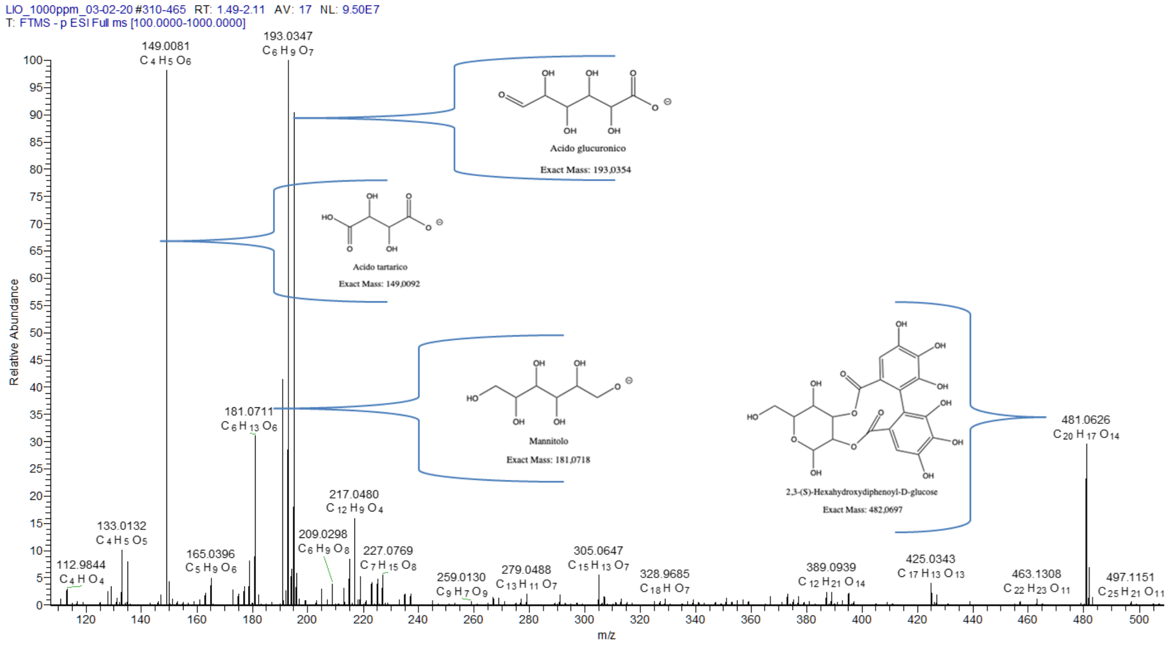

Figure S6. HRMS/MS ESI(-) of peak 1

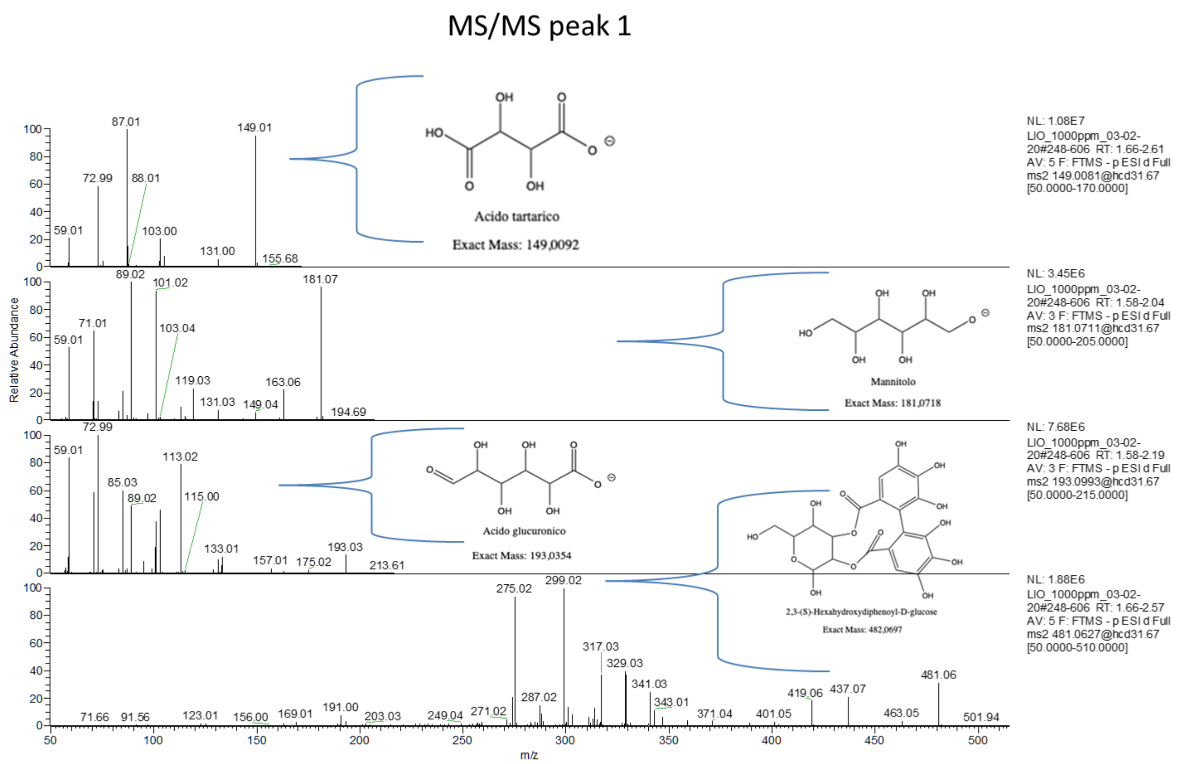

Figure S7. Full LC-HRMS ESI(-) of peak 2

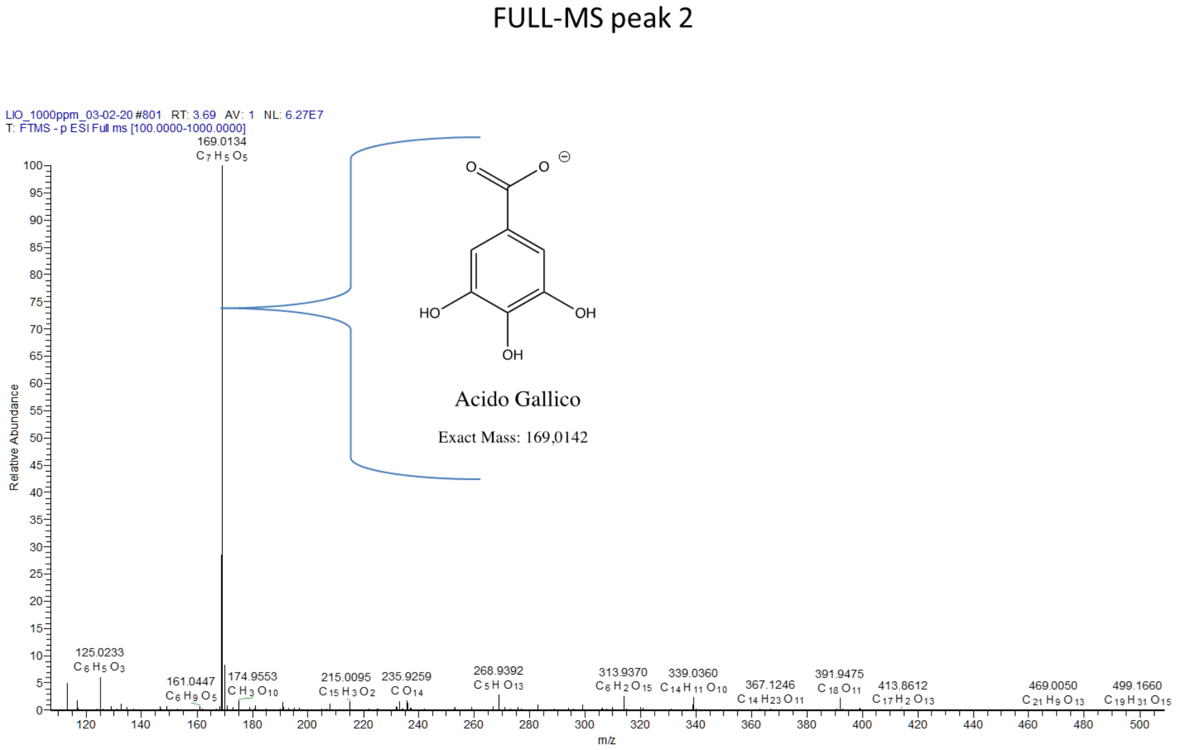

Figure S8. HRMS/MS ESI(-) of peak 2

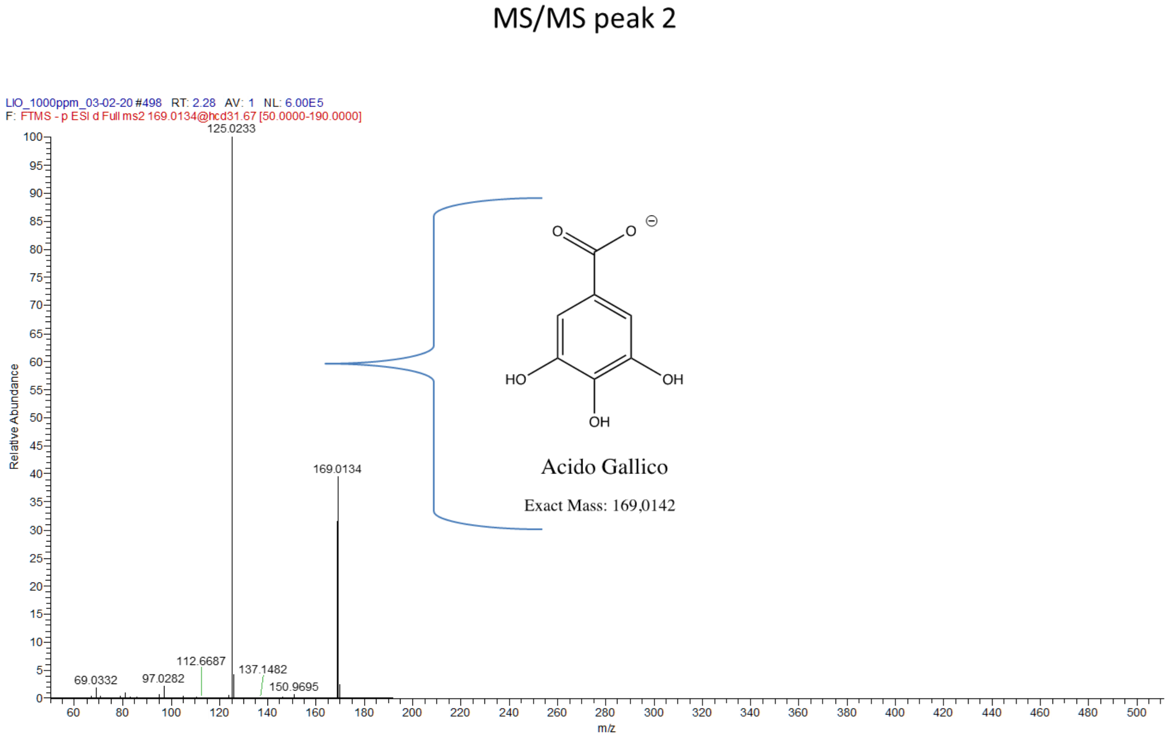

Figure S9. Full LC-HRMS ESI(-) of peak 3

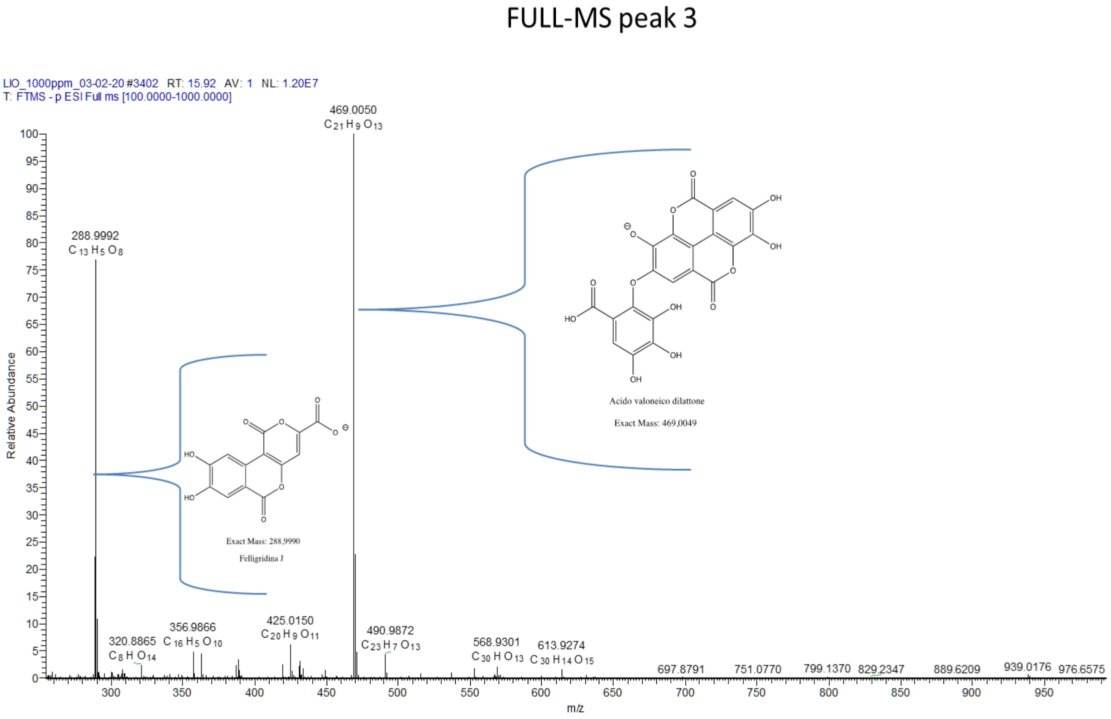

Figure S10. HRMS/MS ESI(-) of peak 3

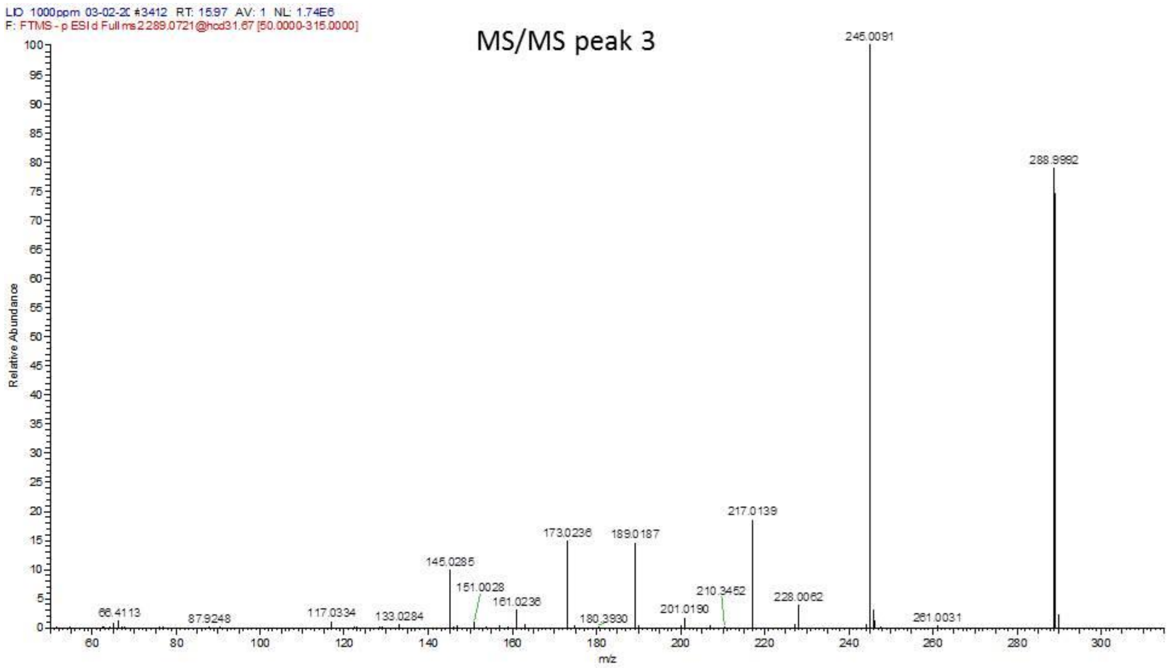

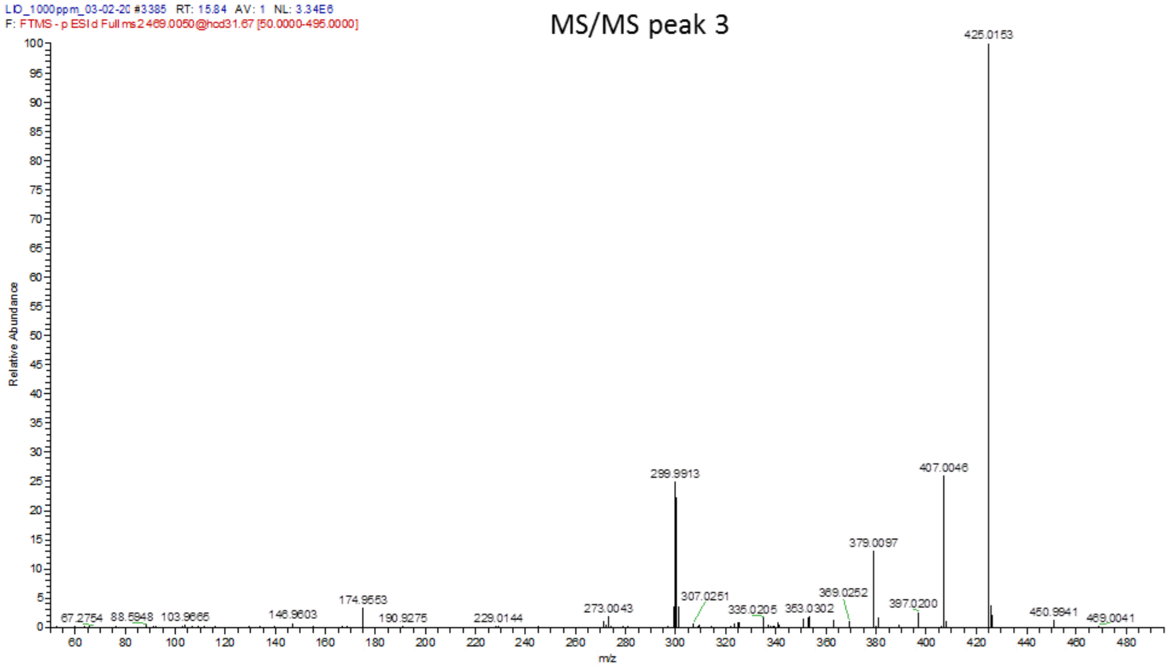

Figure S11. Full LC-HRMS ESI(-) of peak 4

FULL-MS peak 4

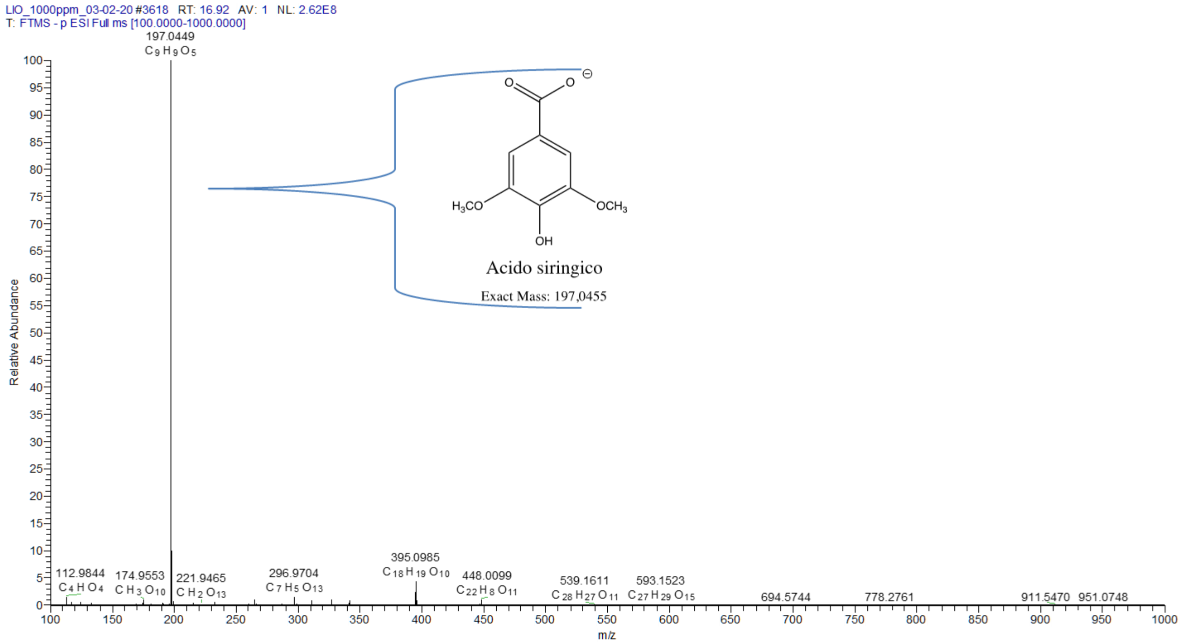

Figure S12. HRMS/MS ESI(-) of peak 4

MS/MS peak 4

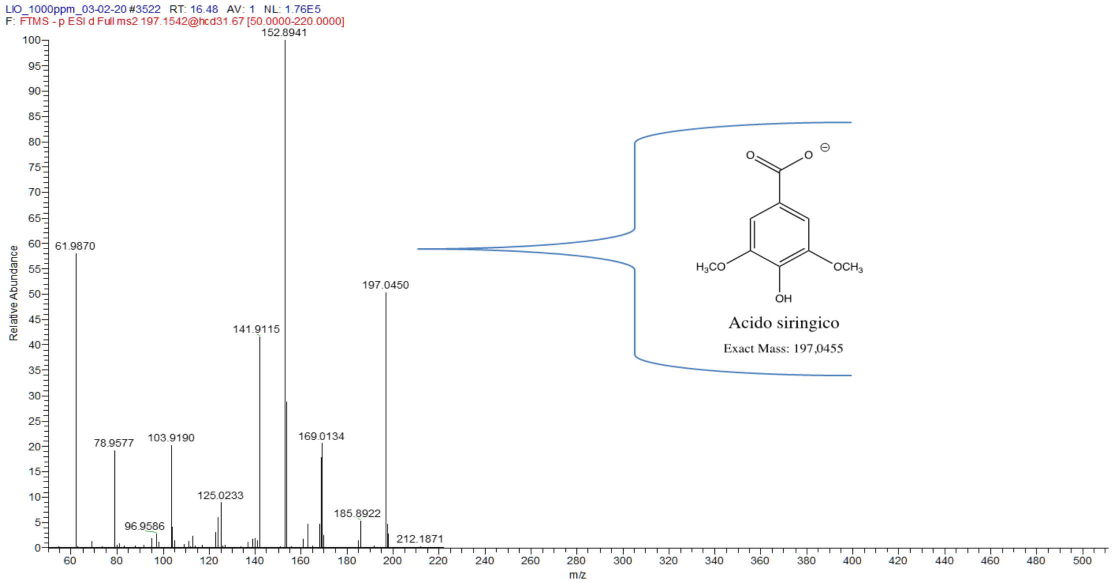

Figure S13. Full LC-HRMS ESI(-) of peak 5

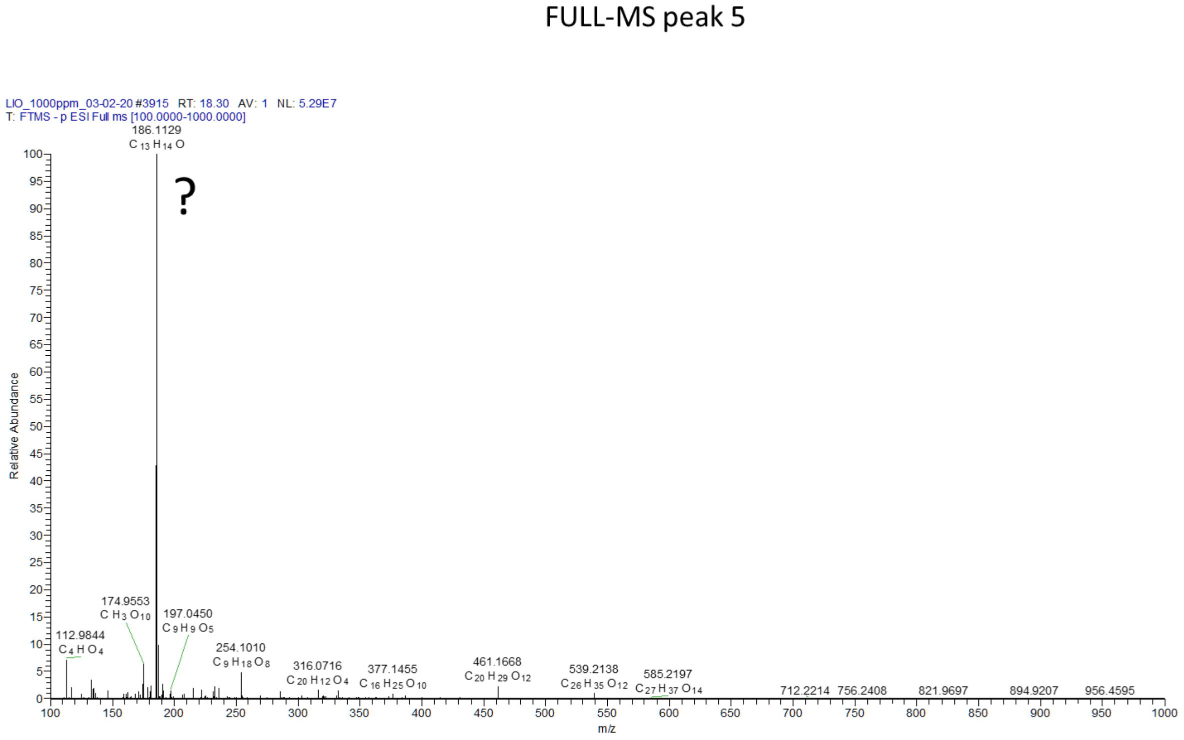

Figure S14. HRMS/MS ESI(-) of peak 5

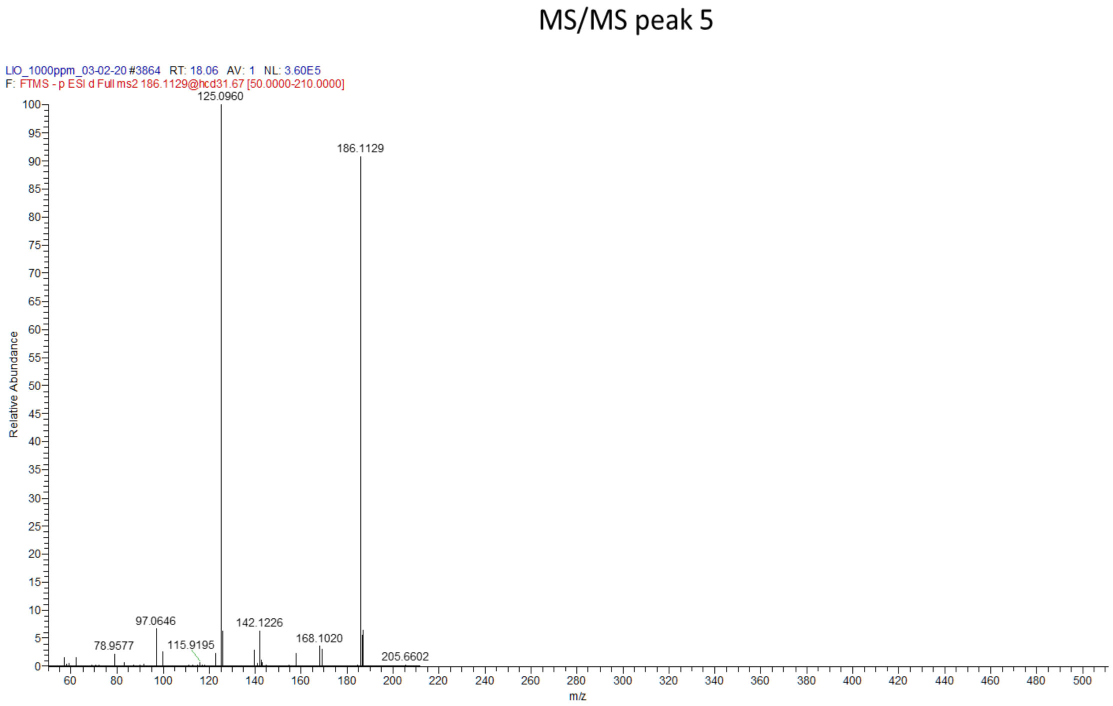

Figure S15. Full LC-HRMS ESI(-) of peak 6

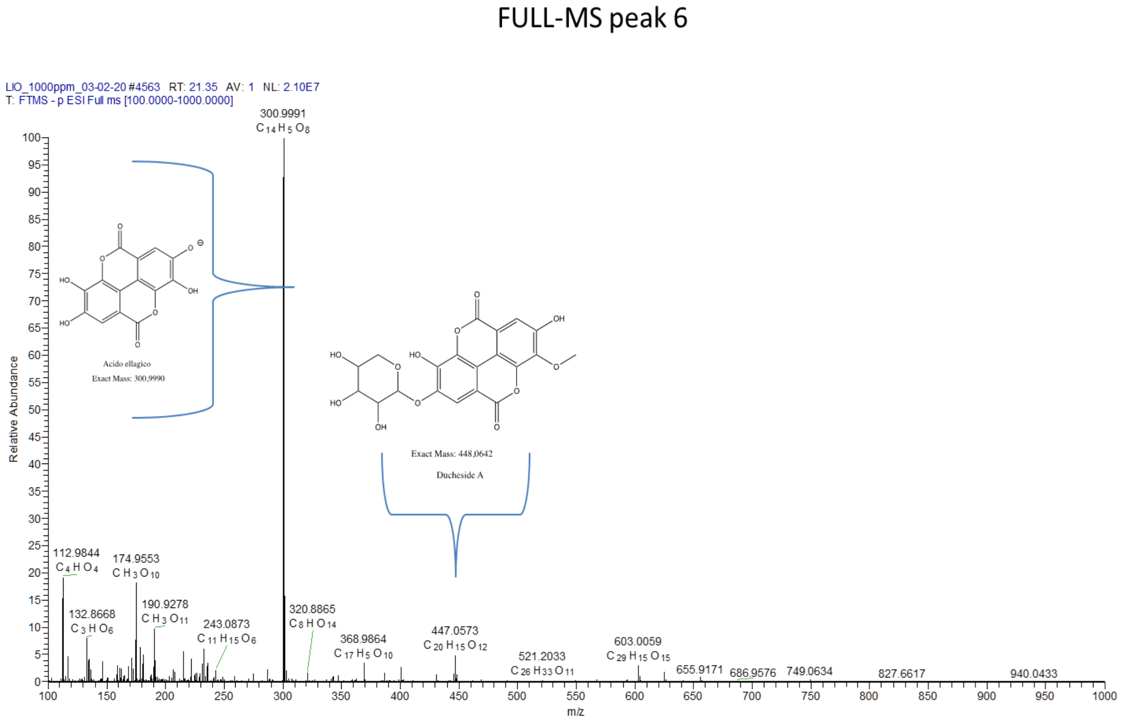

Figure S16. HRMS/MS ESI(-) of peak 6

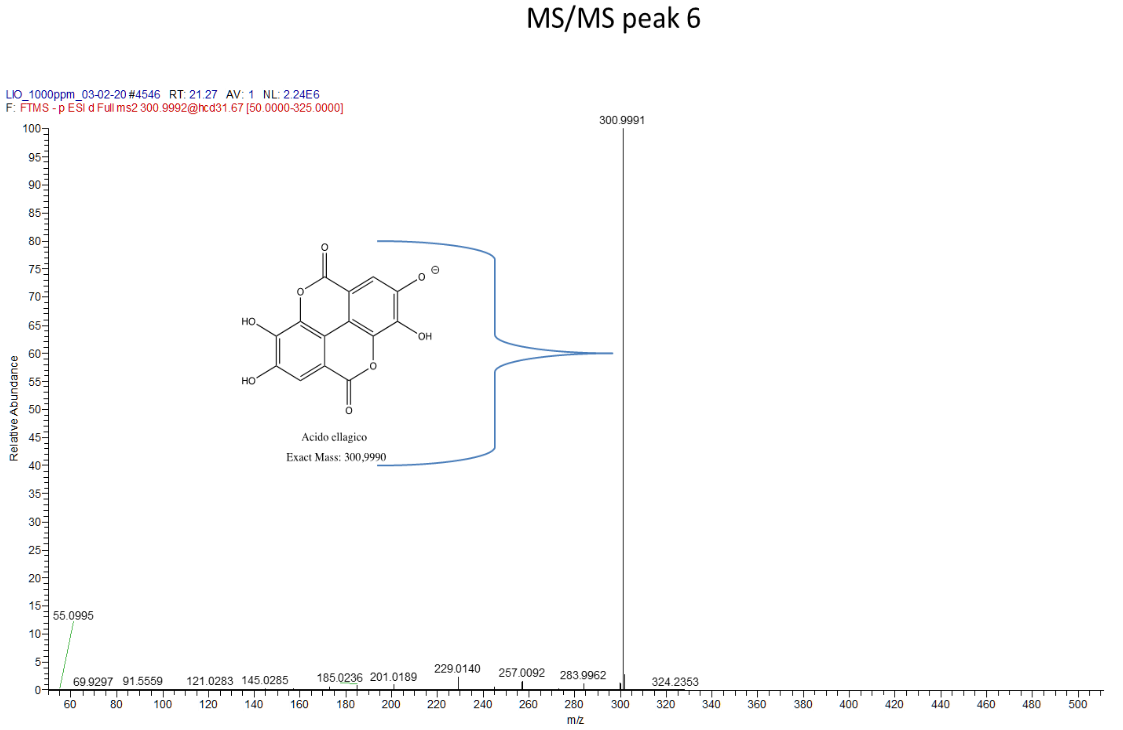

© 2020 by the authors. Licensee MDPI, Basel, Switzerland. This article is an open access article distributed under the terms and conditions of the Creative Commons Attribution (CC BY) license (http://creativecommons.org/licenses/by/4.0/).
